# Handgrip strength—a risk indicator for future fractures in the general population: findings from a prospective study and meta-analysis of 19 prospective cohort studies

**DOI:** 10.1007/s11357-020-00251-8

**Published:** 2020-08-19

**Authors:** Setor K. Kunutsor, Samuel Seidu, Ari Voutilainen, Ashley W. Blom, Jari A. Laukkanen

**Affiliations:** 1 National Institute for Health Research Bristol Biomedical Research Centre, University Hospitals Bristol and Weston NHS Foundation Trust and the University of Bristol, Bristol, UK; 2 Musculoskeletal Research Unit, Translational Health Sciences, Bristol Medical School, University of Bristol, Learning & Research Building (Level 1), Southmead Hospital, Bristol, BS10 5NB UK; 3 Leicester Diabetes Centre, Leicester General Hospital, Gwendolen Road, Leicester, LE5 4WP UK; 4 Diabetes Research Centre, University of Leicester, Leicester General Hospital, Gwendolen Road, Leicester, LE5 4WP UK; 5 Institute of Public Health and Clinical Nutrition, University of Eastern Finland, Kuopio, Finland; 6 Institute of Clinical Medicine, Department of Medicine, University of Eastern Finland, Kuopio, Finland; 7 Central Finland Health Care District Hospital District, Jyväskylä, Finland

**Keywords:** Handgrip strength, Fracture, Cohort study, Meta-analysis

## Abstract

Evolving debate suggests that handgrip strength, a measure of muscular strength, might be associated with the risk of fractures; however, the evidence is conflicting. We aimed to assess the association of handgrip strength with the risk of fracture in the general population. Handgrip strength, measured using a dynamometer, was assessed at baseline in a population-based sample of 853 men and women aged 61–73 years in the Kuopio Ischemic Heart Disease prospective cohort. Hazard ratios (HRs) with 95% confidence intervals (CIs) were estimated for incident fractures. Incident fractures (hip, humeral, or wrist) (*n* = 159) occurred during a median follow-up of 16.7 years. Comparing extreme tertiles of handgrip strength, the age- and sex-adjusted hazard ratio (95% CI) for fractures was 0.80 (0.55–1.18). The association remained similar on further adjustment for other potential confounders: HR (95% CI) of 0.82 (0.55–1.21). In a meta-analysis of 19 population-based prospective cohort studies (including the current study) comprising 220,757 participants and 9199 fractures (including 1302 hip fractures), the fully adjusted relative risk (RR) (95% confidence interval, CI) for incident fractures was 0.70 (0.61–0.80) comparing the top versus bottom thirds of handgrip strength. The association remained significant after trim-and-fill correction for publication bias. The corresponding RR (95% CI) for hip fractures (9 studies) was 0.61 (0.54–0.70). Handgrip was only modestly associated with fracture risk in the primary analysis, which may be driven by the low event rate. Pooled prospective cohort evidence suggests that elevated handgrip strength is associated with reduced future fracture risk.

**Electronic supplementary material:**

The online version of this article (10.1007/s11357-020-00251-8) contains supplementary material, which is available to authorized users.

## Introduction

Fractures (particularly osteoporotic fractures) constitute a global public health burden, especially among older individuals. With the dramatic global demographic shift towards an ageing population, hip fractures alone have been predicted to increase by 35% between 2012 and 2022. Fractures are associated with devastating outcomes such as disability, morbidity, poor quality of life, and mortality. [30, 35] They also pose a major economic burden; the annual hospital cost for treating a hip fracture in the USA is approximately $40,000 and it has been projected that annual fractures and costs will rise by almost 50% by 2025. [7] Ageing, gender, heritability, physical activity, hormonal factors, and nutritional factors such as calcium, vitamin D, and other isolated nutrients such as magnesium play an important role in bone health and the risk of fractures. ((US) [1, 5, 16, 49]) Major risk factors for osteoporotic fractures include reduced bone mass, history of falls, inadequate nutritional absorption, physical inactivity, weight loss, smoking, alcohol consumption, and family history of osteoporosis. [61] The Fracture Risk Assessment Tool (FRAX), the current gold standard tool used in identifying individuals at high risk of hip or other major osteoporotic fracture(10 years), is based on 12 component risk factors including sociodemographic characteristics and medical history as well as femoral neck bone mineral density (BMD).[59] However, it appears some of the component predictors do not explain a large proportion of the risk of fractures, making the identification of individuals at increased risk a difficult undertaking and bringing the reliability of FRAX into question. For example, though low BMD (a proxy for osteoporosis) is a major risk factor for fractures, it has been reported that only a small proportion of people with osteoporosis actually develop fractures. [33] It appears other factors may be involved which could contribute to the residual risk of fractures. From a public health perspective, there is therefore an urgent need to improve upon existing fracture prevention strategies by identifying emerging risk factors which could have predictive relevance for fracture risk. Besides its role in reducing the risk of vascular and other chronic diseases, [17, 46] regular physical activity has also been shown to reduce the risk of fractures. [57, 71] Handgrip strength, commonly used as a typical measure of muscular strength and a proxy for physical fitness, is emerging as a potential risk factor for adverse health outcomes. A number of studies have demonstrated handgrip strength to be inversely associated with adverse vascular and non-vascular outcomes as well as mortality.[10, 18, 42, 52, 53] There is also evidence suggesting an association between high handgrip strength and reduced risk for low bone mass density; [24] however, whether this translates to reduced risk of fractures is uncertain as the existing evidence is divergent. Some studies have shown inverse associations between handgrip strength and fractures, [3, 15, 20, 37] whereas other studies have demonstrated no evidence of an association. [8, 37, 38] The inconsistency in the evidence could be attributed to a number of factors which include differences in sample sizes and follow-up durations; inability of some studies to fully examine the impact of adjustment for potential confounding; differences in study population characteristics such as age, sex, race, or genetic background; differences in handgrip strength assessments; ascertainment and case definition of fracture outcomes; or a combination of all of these. A number of these studies have also been based on case-control designs, [21] which are limited by lack of temporality. There has been a previous attempt to summarize the existing evidence systematically by Denk and colleagues, [21] but this evaluation was limited by the inclusion of a mix of observational cohort and case-control study designs and the authors were unable to conduct a meta-analysis due to the heterogeneous nature of the studies. Whether a prospective relationship exists between handgrip strength and risk of fractures is uncertain. Due to the uncertainty in the evidence, we sought to evaluate in detail the prospective nature of the association between handgrip strength and future fracture risk using a population-based cohort of men and women from eastern Finland followed up for over 20 years. We also performed pooled analysis of available published prospective evidence on the association, thereby offering the opportunity to re-evaluate the nature and magnitude of the association in a larger representative sample of participants and fracture cases.

## Materials and methods

### Study design and population

This study was reported according to STROBE (STrengthening the Reporting of OBservational studies in Epidemiology) guidelines for reporting observational studies in epidemiology (Electronic Supplementary Material 1). Study participants for this analysis were part of the Kuopio Ischemic Heart Disease (KIHD) study, a population-based prospective cohort study designed in Kuopio, Finland, to investigate risk factors for cardiovascular disease (CVD) and other related chronic diseases. [45, 66] In the original KIHD study, participants were a representative sample of men aged 42–61 years recruited from Kuopio city and its surrounding rural communities in eastern Finland. These participants underwent re-examinations at 4 years, 11 years, and 20 years after study entry. At the 11-year follow-up re-examinations, a randomly selected group of women aged 53–74 years were invited to join the original study and they formed part of the cohort employed for this analysis. The cohort utilized for the current analysis initially comprised 2358 participants (1007 men and 1351 women) who were aged 53 to 74 years at study entry. [41] Of the 2072 participants found to be potentially eligible, 193 did not agree to participate, 66 did not respond to the invitation, and 39 declined to provide informed consent, which left 1774 participants. [41] Baseline examinations were conducted from March 1998 to December 2001. [41] A subset of 875 randomly selected participants had handgrip strength measurements at the 11-year re-examination (baseline examination for this cohort). Of the 1774 eligible participants, we excluded 921 participants with incomplete data on handgrip strength and relevant covariates (of which 899 did not have data on handgrip strength measurements). For the current analysis, 853 men and women had non-missing information on handgrip strength, potential confounders, and fracture outcomes (Electronic Supplementary Material 2).

### Ethics

The research protocol was approved by the institutional review board of the University of Kuopio and Kuopio University Hospital, Kuopio, Finland (License number 143/97). All study procedures were conducted according to the Declaration of Helsinki and written informed consent was obtained from all participants

### Assessment of handgrip strength and relevant risk markers

Handgrip strength of the dominant hand for each participant was measured by a hand dynamometer (in kPa; Martin-Balloon-Vigorimeter; Gebrüder Martin, Tuttlingen, Germany); two measurements were taken, and the mean of both values was used for analysis. [42, 47, 52, 53] The dynamometers were calibrated at the beginning of each test and there was a 1-min resting gap between both handgrip measurements. Study procedures including blood sample collection, physical measurements, assessment of lifestyle characteristics, and measurement of blood-based markers have been described previously. [41, 44] Participants fasted overnight and abstained from drinking alcohol for at least 3 days and smoking for at least 12 h before blood collection. Blood lipids were measured enzymatically (Boehringer Mannheim, Mannheim, Germany) from fresh serum samples after combined ultracentrifugation and precipitation. [43] Serum high-sensitivity C-reactive protein (hsCRP) measurements were made with an immunometric assay (Immulite High Sensitivity C-Reactive Protein Assay; DPC, Los Angeles, CA, USA). Self-reported questionnaires were used to assess baseline sociodemographic and lifestyle characteristics, existing medical conditions, and use of medications. [55] The energy expenditure of physical activity was assessed from a validated 12-month leisure-time physical activity questionnaire. [51] Body mass index (BMI) was calculated by dividing weight measured in kilograms by the square of height in meters.

### Ascertainment of incident fractures

All incident fractures (defined as hip, humeral, or wrist fractures) cases that occurred from study entry to 2017 were included. In the KIHD study, participants are under annual continuous surveillance for the development of new outcome events (including fractures) and no losses to follow-up have been recorded. Data on incident fractures was collected from the National Hospital Discharge Register data by computer linkage using Finnish personal identification codes and a comprehensive review of hospital records, discharge diagnoses, and inpatient physician claims. Fracture outcomes were coded according to the International Classification of Diseases Tenth Revision diagnostic codes for fractures by site.

### Statistical analyses

#### Prospective cohort analyses

Baseline characteristics were presented as means (standard deviation, SD) or medians (interquartile range, IQR) for continuous variables and percentages for categorical variables using descriptive analyses. Hazard ratios (HRs) with 95% confidence intervals (CIs) for fractures were calculated using Cox proportional hazard models after confirmation of no major departure from the proportionality of hazard assumptions using Schoenfeld residuals. Handgrip strength was modeled as a continuous (per standard deviation (SD) increase) and categorical (tertiles) exposure variable. Hazard ratios were adjusted for in two models: (i) age and sex and (ii) plus BMI, smoking status, prevalent coronary heart disease (CHD), history of type 2 diabetes mellitus, physical activity, and hsCRP.

#### Systematic review and meta-analysis

We conducted a meta-analysis of published observational cohort studies reporting on the association between handgrip strength and risk of fractures, using a predefined protocol which was reported in accordance with PRISMA and MOOSE guidelines [58, 72] (Electronic Supplementary Materials 3-4). Published observational population-based cohort (prospective cohort, retrospective cohort, case cohort, or nested case-control) studies that evaluated the associations between baseline values of handgrip strength and risk of fracture up to 01 April 2020 were searched for using computer-based databases (MEDLINE, EMBASE, and the “Cited Reference Search” function in Web of Science). The computer-based searches combined free and MeSH search terms or key words related to the exposure (e.g., “handgrip strength,” “muscle strength”) and outcome (e.g., “fracture”). No restrictions were placed on language or date of publication. The detailed search strategy is reported in Electronic Supplementary Material 5. The summary measure of association was presented as a relative risk (RR) with 95% confidence intervals (CI). Hazard ratios and odds ratios were assumed to approximate the same measure of RR following Cornfield’s rare disease assumption. [19] To enable a consistent approach to the meta-analysis and enhance pooling and comparison with the primary analysis, reported study-specific risk estimates were transformed to comparisons involving the top versus bottom tertiles of handgrip strength values using standard statistical methods, [14, 29] which have been described in previous reports. [39, 40] For comparisons that could not be transformed, the extreme groups (i.e., maximum versus minimal value of handgrip strength) were used for the analyses, as reported previously. [46] When the highest handgrip strength was the referent, we converted the reported risk estimate into its reciprocal. Risk estimates were pooled using a random effects model to minimize the effect of between-study heterogeneity. [22] Between-study statistical heterogeneity was quantified using standard chi-square tests and the *I*^2^ statistic. [32] We also assessed the potential for small study effects such as publication bias through formal tests, namely Begg’s funnel plots [4] and Egger’s regression symmetry test. [26] Finally, we adjusted for the effect of publication bias by the use of the Duval and Tweedie’s nonparametric trim-and-fill method which imputes hypothetical small missing null or negative studies. [25] All statistical analyses were performed using Stata version MP 16 (Stata Corp, College Station, Texas).

## Results

### Baseline characteristics

The overall mean (SD) age of study participants at baseline was 69 (3) years and 47.4% comprised of males. The mean (SD) handgrip strength at baseline was 76.2 (21.1) kPa (Table 1). During a median (IQR) follow-up of 16.7 (10.8–18.2) years, a total of 159 fractures (annual rate 13.23/1000 person-years at risk; 95% CI: 11.33 to 15.46) were recorded and these included 69 hip fractures (annual rate 5.45/1000 person-years at risk; 95% CI: 4.30 to 6.90).

**Table 1:** Baseline participant characteristics

	Mean (SD), median (IQR), or *n* (%)
Handgrip strength (kPa)	76.2 (21.1)
Questionnaire/prevalent conditions	
Age at survey (years)	69 (3)
Males	404 (47.4)
History of type 2 diabetes	81 (9.5)
Current smokers	81 (9.5)
History of CHD	306 (35.9)
Physical measurements	
BMI (kg/m^2^)	27.9 (4.3)
SBP (mmHg)	138 (18)
DBP (mmHg)	80 (9)
Energy expenditure of total LTPA (kcal/day)	378 (227–652)
Blood-based markers	
Total cholesterol (mmol/l)	5.45 (0.94)
HDL-C (mmol/l)	1.24 (0.32)
High-sensitivity CRP	1.60 (0.79–3.22)

*BMI*, body mass index; *CHD*, coronary heart disease; *CI*, confidence interval; *CRP*, C-reactive protein; *DBP*, diastolic blood pressure; *HDL-C*, high-density lipoprotein cholesterol; *IQR*, interquartile range; *LTPA*, leisure-time physical activity; *SD*, standard deviation; *SBP*, systolic blood pressure

### Handgrip strength and fracture risk

#### Prospective cohort analysis

The age- and sex-adjusted HR for fracture per 1 SD increase in handgrip strength was 0.93 (95% CI: 0.78–1.12) which was minimally attenuated to 0.95 (95% CI: 0.80–1.13) on further adjustment for several established risk factors and other potential confounders (BMI, smoking status, prevalent CHD, history of type 2 diabetes mellitus, physical activity, and hsCRP). Alternatively, comparing the top versus bottom tertile of handgrip strength values, the corresponding adjusted HRs were 0.80 (95% CI: 0.55–1.18) and 0.82 (95% CI: 0.55–1.21), respectively (Table 2). Comparing the top versus bottom third of handgrip strength, the fully adjusted HRs for fractures in men and women were 0.59 (95% CI: 0.29–1.22) and 0.85 (95% CI: 0.53–1.37), respectively. The non-significant associations persisted in analysis limited to hip fracture (Table 2). To put the strength of the association of handgrip strength with fracture risk into context, the associations of risk factors within the KIHD cohort with fracture risk were compared. Age, gender, and physical activity were associated with the risk of both total and hip fractures (Electronic Supplementary Material 6).

**Table 2:** Associations of handgrip strength with fractures

Handgrip strength (kPa)	Events/total	Model 1		Model 2	
		HR (95% CI)	*p* value	HR (95% CI)	*p* value
A.1.1.1.1.1.1.1. Total fractures					
Per 1 SD increase	159/853	0.93 (0.78−1.12)	.44	0.95 (0.80−1.13)	.55
Tertile 1 (0.27−0.91)	64/286	1 [Reference]		1 [Reference]	
Tertile 2 (0.92−1.11)	49/283	0.73 (0.51−1.07)	.10	0.77 (0.53−1.13)	.18
Tertile 3 (1.12−7.31)	46/284	0.80 (0.55−1.18)	.27	0.82 (0.55−1.21)	.32
A.1.1.1.1.1.1.2. Hip fractures					
Per 1 SD increase	69/853	0.99 (0.77−1.28)	.96	0.99 (0.77−1.28)	.96
Tertile 1 (0.27−0.91)	29/286	1 [Reference]		1 [Reference]	
Tertile 2 (0.92−1.11)	18/283	0.61 (0.34−1.10)	.10	0.63 (0.35−1.15)	.14
Tertile 3 (1.12−7.31)	22/284	0.98 (0.56−1.74)	.95	0.95 (0.53−1.71)	.88

*SD*, standard deviation

Model 1: adjusted for age and gender

Model 2: model 1 plus body mass index, smoking status, prevalent coronary heart disease, history of type 2 diabetes mellitus, physical activity, and high-sensitivity C-reactive protein

#### Meta-analysis of published studies

**Fig. 1:**
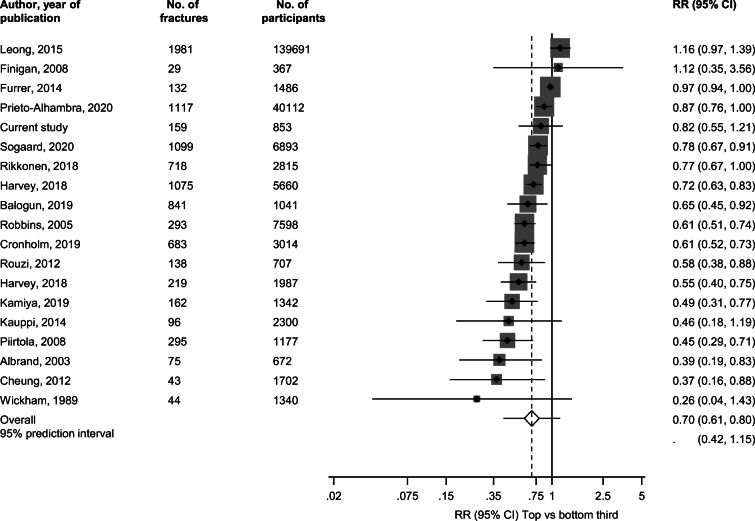
Associations between handgrip strength and risk of fractures in prospective cohort studies. The summary estimates presented were calculated using random effects models; relative risks are reported comparing extreme tertiles of handgrip strength; size of data markers is proportional to the inverse of the variance of the relative ratio; CI, confidence interval (bars); RR, relative risk

**Fig. 2:**
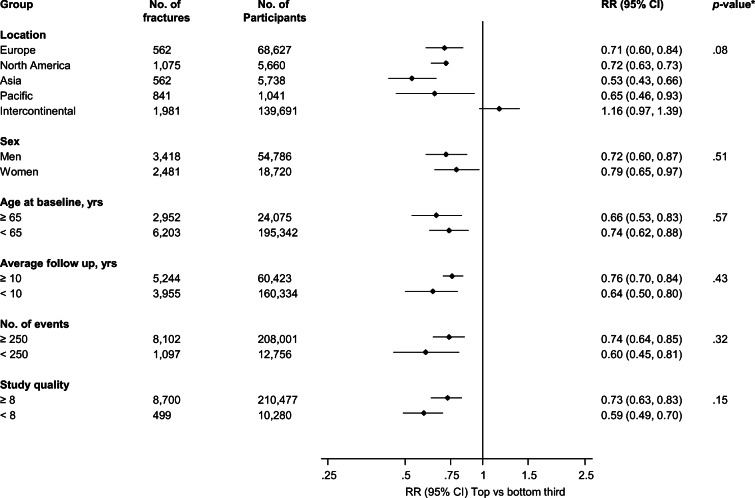
Associations between handgrip strength and risk of fractures by several study level characteristics. The summary estimates presented were calculated using random effects models; CI, confidence interval (bars); RR, relative risk; *p value for meta-regression

We identified 18 population-based prospective cohort studies reporting on the associations between handgrip strength and fracture risk (Electronic Supplementary Material 7 and Table 3). [2, 3, 15, 20, 27, 28, 31, 36, 38, 54, 60, 62–65, 70, 74] Including the current study, the pooled analysis comprised 19 studies involving 220,757 participants and 9199 incident fractures (comprising 1302 hip fractures). Average age of participants ranged from 18 to 81 years with a weighted mean (SD) of 48 (16) years. Average follow-up duration ranged from 2.9 to 18.3 years with a weighted mean (SD) of 7.7 years. Comparing the top versus bottom tertile of handgrip strength values, the pooled RR for incident fractures was 0.70 (95% CI: 0.61–0.80) (Fig. 1). The 95% prediction interval for the pooled RR was 0.42 to 1.15%, suggesting that the true RR for any single new study will usually fall within this range. There was evidence of substantial heterogeneity between the contributing studies (*I*^2^ = 86%, 79 to 90%; *p* < 0.001), which was not explained by any of the study level characteristics prespecified for subgroup analysis (Fig. 2). A funnel plot of the contributing studies showed visual evidence of asymmetry (Electronic Supplementary Material 8) which was consistent with Egger’s regression symmetry test (*p* < 0.001). The trim-and-fill technique, which was used to adjust for publication bias, imputed five missing studies which produced a symmetrical funnel plot (Electronic Supplementary Material 9). In analysis that incorporated the hypothetical studies, the pooled RR for incident fractures was similar 0.74 (95% CI: 0.65–0.85).

**Table 3:** Baseline characteristics of eligible prospective cohort studies (1989–2020)

Author, year of publication	Study name/source	Country	Baseline year	Average age (years)	Males (%)	Follow-up (years)	No. of fractures	Number of participants	Quality score
Wickham, 1989	NR	UK	1973−1974	≥ 65	NR	15.0	44	1340	7
Albrand, 2003	OFELY Study	France	1992−1993	59.1	0	5.3	75	672	8
Robbins, 2005	EPIDOS	France	1992−1994	80.5	0	3.0	293	7598	6
Cawthon, 2008	MrOS	USA	2000−2002	≥ 65	100	5.3	77	5902	7
Finigan, 2008	NR	UK	1990−1991	64.6	0	10.0	29	367	8
Piirtola, 2008	NR	Finland	1990−1991	73.0	41	8.5	295	1177	8
Cheung, 2012	HKOS	China	1998−2009	64.1	51.8	2.9	43	1702	8
Rouzi, 2012	CEOR	Saudi Arabia	2004	61.3	0	5.2	138	707	8
Kauppi, 2014	Health 2000 Survey	Finland	2000−2001	66.4	42.1	9.8	96	2300	9
Furrer, 2014	LASA	Netherlands	1995−1996; 1998−1999	75.8	48	6.0	132	1486	8
Leong, 2015	PURE	17 countries	2003−2009	50.0	42	4.0	1,981	139,691	9
Harvey, 2018	MrOS USA	USA	2000−2003	73.5	100	10.9	1,075	5660	8
Harvey, 2018	MrOS Hong Kong	Hong Kong	2001−2003	72.4	100	9.9	219	1987	8
Rikkonen, 2018	OSTPRE	Finland	1989−1994	59.1	0	18.3	261	2815	8
Balogun, 2019	TASOAC	Australia	2002−2004	63.0	50	10.0	841	1041	8
Cronholm, 2019	MrOS Sweden	Sweden	2001−2004	75.4	100	9.6	683	3014	8
Kamiya, 2019	JPOCS	Japan	1996	63.4	0	15.2	162	1342	7
Prieto-Alhambra, 2020	NPR	Sweden	1969−1970	18.0	100	18.1	1,117	40,112	8
Sogaard, 2020	Tromso	Norway	1994−1995	61.8	41.9	15.0	1,099	6893	9
Current study	KIHD	Finland	1998−2001	69.0	47.4	16.7	159	853	9

*CEOR*, Center of Excellence for Osteoporosis Research; *EPIDOS*, Epidémiologie de l’ostéoporose; *HKOS*, Hong Kong Osteoporosis Study; *KIHD*, Kuopio Ischemic Heart Disease; *JPOCS*, Japanese Population-based Osteoporosis Cohort Study; *LASA*, Longitudinal Aging Study Amsterdam; *MrOS*, Osteoporotic Fractures in Men; *NPR*, National Patient Register; *OSTPRE*, Osteoporosis Risk Factor and Prevention Study; *NR*, not reported; *PURE*, Prospective Urban-Rural Epidemiology; *TASOAC*, Tasmanian Older Adult Cohort

In pooled analysis of 9 studies that contributed hip fracture data, the pooled RR for incident hip fractures was 0.61 (95% CI: 0.54–0.70) (Electronic Supplementary Material 10).

## Discussion

Our analysis of a population-based prospective study of older Finnish men and women demonstrated no evidence of an association between handgrip strength and risk of future risk of fractures, which could be due to inadequate power given the low number of fracture events. We however confirmed existing evidence on the associations of fracture risk with age, gender, and physical activity. [61] To re-evaluate the findings between handgrip strength and fracture risk, we pooled all available prospective studies published on the topic and we found strong evidence of an association between elevated handgrip strength and the reduced risk of fractures. Though there was evidence of small study effects, the association persisted after correction for publication bias using trim-and-fill techniques. The association of handgrip strength with fracture risk was not modified by relevant study characteristics such as age, gender, and location. In subsidiary analyses limited to hip fractures, there was still evidence of a significant association.

Findings on the relationship between handgrip strength and the risk of fractures have been divergent in the absence of a pooled analysis. To the best of our knowledge, there have been no previous attempts to quantitatively aggregate existing data on the association between handgrip strength and the risk of fractures. Denk and colleagues in a systematic review of 11 studies comprising 6 case-control and 5 cohort studies concluded there was an association between decreased handgrip strength and low impact hip fracture. [21] The authors were unable to pool the evidence quantitatively due to heterogeneity; their conclusion was based on the observation that all 11 studies reported handgrip strength to be associated with hip fracture risk. A major limitation of this approach is drawing conclusions from case-control designs, which are characterized by selection bias and their inability to address temporality. By pooling 19 available prospective studies which provided enhanced power, we have been able to show that increased handgrip strength is associated with a reduced future risk of any as well as hip fractures.

It has been postulated that handgrip strength may be linked to the risk of fractures via ageing, frailty, sarcopenia, and osteoporosis, which are all major risk factors for fractures. Ageing is generally associated with the development of chronic disease conditions as well as frailty, sarcopenia, and osteoporosis. Decreased handgrip strength is associated with frailty, [6] which is often associated with fatigue, reduced muscle mass and strength, and high susceptibility to falls. Sarcopenia, which is an important aspect of frailty [34] and a major risk factor for hip fractures, [73] is characterized by loss of muscle mass and strength, neurodegeneration, and poor physical performance, [13] leading to decreased bone strength and increased risk of falls. [9]

Handgrip strength is well known to be an independent risk marker for vascular disease and has been demonstrated to be of value in the discrimination and reclassification of individuals at risk for CVD. [11, 46] The current findings suggest that handgrip strength may also be a strong risk indicator for incident fractures. Testing handgrip strength could be used as a quick, low-cost screening tool to help healthcare professionals identify patients at risk of fractures. We have recently shown that handgrip strength improves the prediction of CVD mortality as well as type 2 diabetes beyond conventional risk factors. ([48] (In Press); [52]) Early assessment of handgrip strength in fracture patients at hospital admission has also been shown to independently predict functional outcomes. [23, 67, 68] The potential inclusion of handgrip strength in well-known fracture risk assessment tools such as FRAX and its ability to provide prognostic information on the functional trajectory of fracture patients warrants evaluation. Large-scale studies with relevant data are needed to evaluate the specificity of the association and investigate the applicability of handgrip strength in fracture prevention.

## Strengths and limitations

Strengths of the current study include the well-characterized cohort of men and women who were nationally representative in the age group considered; use of the MartinVigorimeter, known for its high reliability and accuracy, in assessing grip strength especially in older patients; [69] long-follow-up duration of the cohort with no losses to follow-up recorded; and the comprehensive analyses. Furthermore, we were able to conduct a pooled analysis of previous cohort studies including the current study, to put the findings into wider context. This analysis had enhanced power to reliably assess the nature and magnitude of the association between handgrip strength and fracture risk. We were able to extract and analyze data on hip-specific fractures. Our analysis was comprehensive which included exploration of sources of heterogeneity and testing and adjusting for publication bias. Though a comprehensive search of the major databases was conducted to identify all previous studies on the topic, formal tests seemed to suggest evidence of publication bias; however, adjustment for publication bias did not alter the observed results. There were important limitations to this review and these were all inherent to our primary data and studies included in the meta-analysis. Our primary analysis was based in older predominantly white-European men and women from eastern Finland; hence, the findings cannot be generalized to younger age groups and other ethnicities. We only had outcome data on fractures related to the hip, humerus, and wrist and therefore could not assess the associations for a broad range of fractures related to osteoporosis, such as radial and vertebral crush fractures; we also did not have any data on subtypes of wrist fractures. Though we adjusted for a comprehensive panel of risk factors, other potential confounders that could not be accounted for included prevalent conditions (e.g., Crohn’s disease, coeliac disease, thyroid disease, myelomas, renal disease, liver disease), previous fracture, use of medications, and other factors associated with bone health such as parathyroid hormone and vitamin D levels. Vitamin D insufficiency is common in northern countries such as Finland during the winter because of inadequate exposure to sunlight during that period; it is quite commonplace among all age groups in Finland. [50] Due to absence of repeat measurements of handgrip strength, we could not correct for regression dilution bias; [56] this potentially results in the underestimation of the true association between an exposure and outcome, particularly for cohorts with long-term follow-up due to measurements errors, lifestyle changes, ageing, and chronic disease. Furthermore, we could not account for management strategies of osteoporosis, which might have likely affected the incidence of fractures and impacted our estimates. We acknowledge the potential for selection bias given that study participants provided informed consent and this may potentially bias the results; however, this is an inherent limitation of observational cohort designs. In the pooled analysis, a few of the risk estimates could not be transformed into tertiles; hence, comparisons could only be made between the maximum versus minimum value of handgrip strength. However, we have demonstrated in a previous study that pooled results from untransformed data of extreme categories are not very different from results based on transformed data. [12] There was substantial heterogeneity between contributing studies which could not be explained by several clinically relevant study level characteristics, suggesting other sources of variation such as participants (age and sex), assessment methods for handgrip strength, ascertainment of outcomes, and in the results, which can only be explored with access to individual level data.

## Conclusion

Handgrip was only modestly associated with fracture risk in the primary analysis, which may be driven by the low event rate. Pooled prospective cohort evidence suggests that elevated handgrip strength is associated with reduced future fracture risk.

## Electronic supplementary material

ESM 1 (DOCX 328 kb)

## References

[1] (US) OotSG (2004). Bone health and osteoporosis: a report of the surgeon general. Reports of the Surgeon General.

[2] Albrand G, Munoz F, Sornay-Rendu E, DuBoeuf F, Delmas PD (2003). Independent predictors of all osteoporosis-related fractures in healthy postmenopausal women: the OFELY study. Bone.

[3] Balogun S (2019). Prospective associations of osteosarcopenia and osteodynapenia with incident fracture and mortality over 10 years in community-dwelling older adults. Arch Gerontol Geriatr.

[4] Begg CB, Mazumdar M (1994). Operating characteristics of a rank correlation test for publication bias. Biometrics.

[5] Bischoff-Ferrari HA (2012). A pooled analysis of vitamin D dose requirements for fracture prevention. N Engl J Med.

[6] Bohannon RW (2019). Grip strength: an indispensable biomarker for older. Adults Clin Interv Aging.

[7] Burge R, Dawson-Hughes B, Solomon DH, Wong JB, King A, Tosteson A. Incidence and economic burden of osteoporosis-related fractures in the United States, 2005-2025. J Bone Miner Res. 2007;22:465–75. 10.1359/jbmr.061113. 10.1359/jbmr.061113 17144789

[8] Cawthon PM, Fullman RL, Marshall L, Mackey DC, Fink HA, Cauley JA, Cummings SR, Orwoll ES, Ensrud KE, Osteoporotic Fractures in Men (MrOS) Research Group (2008). Physical performance and risk of hip fractures in older men. J Bone Miner Res.

[9] Cederholm T, Cruz-Jentoft AJ, Maggi S (2013). Sarcopenia and fragility fractures. Eur J Phys Rehabil Med.

[10] Celis-Morales CA (2017). The association between physical activity and risk of mortality is modulated by grip strength and cardiorespiratory fitness: evidence from 498 135 UK-Biobank participants. Eur Heart J.

[11] Celis-Morales CA (2018). Associations of grip strength with cardiovascular, respiratory, and cancer outcomes and all cause mortality: prospective cohort study of half a million UK Biobank participants. BMJ.

[12] Chen HG (2019). Association of vitamin K with cardiovascular events and all-cause mortality: a systematic review and meta-analysis. Eur J Nutr.

[13] Chen LK (2014). Sarcopenia in Asia: consensus report of the Asian Working Group for Sarcopenia. J Am Med Dir Assoc.

[14] Chêne G, Thompson SG (1996). Methods for summarizing the risk associations of quantitative variables in epidemiologic studies in a consistent form. Am J Epidemiol.

[15] Cheung CL, Tan KC, Bow CH, Soong CS, Loong CH, Kung AW (2012). Low handgrip strength is a predictor of osteoporotic fractures: cross-sectional and prospective evidence from the Hong Kong Osteoporosis Study. Age (Dordr).

[16] Chung M, Lee J, Terasawa T, Lau J, Trikalinos TA (2011). Vitamin D with or without calcium supplementation for prevention of cancer and fractures: an updated meta-analysis for the U.S. Preventive Services Task Force. Ann Intern Med.

[17] Colpani V (2018). Lifestyle factors, cardiovascular disease and all-cause mortality in middle-aged and elderly women: a systematic review and meta-analysis. Eur J Epidemiol.

[18] Cooper R, Kuh D, Hardy R, Mortality Review G, Falcon, Teams HAS (2010). Objectively measured physical capability levels and mortality: systematic review and meta-analysis. BMJ.

[19] Cornfield J (1951). A method of estimating comparative rates from clinical data; applications to cancer of the lung, breast, and cervix. J Natl Cancer Inst.

[20] Cronholm F, Rosengren BE, Nilsson JA, Ohlsson C, Mellstrom D, Ribom E, Karlsson MK (2019). The fracture predictive ability of a musculoskeletal composite score in old men - data from the MrOs Sweden study. BMC Geriatr.

[21] Denk K, Lennon S, Gordon S, Jaarsma RL (2018). The association between decreased hand grip strength and hip fracture in older people: a systematic review. Exp Gerontol.

[22] DerSimonian R, Laird N (1986). Meta-analysis in clinical trials. Control Clin Trials.

[23] Di Monaco M, Castiglioni C, De Toma E, Gardin L, Giordano S, Tappero R (2015). Handgrip strength is an independent predictor of functional outcome in hip-fracture women: a prospective study with 6-month follow-up. Medicine (Baltimore).

[24] Dixon WG (2005). Low grip strength is associated with bone mineral density and vertebral fracture in women. Rheumatology (Oxford).

[25] Duval S, Tweedie R (2000). Trim and fill: a simple funnel-plot-based method of testing and adjusting for publication bias in meta-analysis. Biometrics.

[26] Egger M, Davey Smith G, Schneider M, Minder C (1997). Bias in meta-analysis detected by a simple, graphical test. BMJ.

[27] Finigan J, Greenfield DM, Blumsohn A, Hannon RA, Peel NF, Jiang G, Eastell R (2008). Risk factors for vertebral and nonvertebral fracture over 10 years: a population-based study in women. J Bone Miner Res.

[28] Furrer R, van Schoor NM, de Haan A, Lips P, de Jongh RT (2014). Gender-specific associations between physical functioning, bone quality, and fracture risk in older people. Calcif Tissue Int.

[29] Greenland S, Longnecker MP (1992). Methods for trend estimation from summarized dose-response data, with applications to meta-analysis. Am J Epidemiol.

[30] Griffin XL, Parsons N, Achten J, Fernandez M, Costa ML (2015). Recovery of health-related quality of life in a United Kingdom hip fracture population. The Warwick Hip Trauma Evaluation--a prospective cohort study. Bone Joint J.

[31] Harvey NC (2018). Measures of physical performance and muscle strength as predictors of fracture risk independent of FRAX, falls, and aBMD: a meta-analysis of the osteoporotic fractures in men (MrOS) Study. J Bone Miner Res.

[32] Higgins JP, Thompson SG, Deeks JJ, Altman DG (2003). Measuring inconsistency in meta-analyses. BMJ.

[33] Jarvinen TL, Michaelsson K, Jokihaara J, Collins GS, Perry TL, Mintzes B, Musini V, Erviti J, Gorricho J, Wright JM, Sievanen H (2015). Overdiagnosis of bone fragility in the quest to prevent hip fracture. BMJ.

[34] Jin X (2020). Epidemiological, clinical and virological characteristics of 74 cases of coronavirus-infected disease 2019 (COVID-19) with gastrointestinal symptoms. Gut.

[35] Johnell O, Kanis JA (2006). An estimate of the worldwide prevalence and disability associated with osteoporotic fractures. Osteoporos Int.

[36] Kamiya K (2019). Association between hand-grip strength and site-specific risks of major osteoporotic fracture: results from the Japanese Population-based Osteoporosis Cohort Study. Maturitas.

[37] Karkkainen M (2008). Association between functional capacity tests and fractures: an eight-year prospective population-based cohort study. Osteoporos Int.

[38] Kauppi M, Stenholm S, Impivaara O, Maki J, Heliovaara M, Jula A (2014). Fall-related risk factors and heel quantitative ultrasound in the assessment of hip fracture risk: a 10-year follow-up of a nationally representative adult population sample. Osteoporos Int.

[39] Kunutsor SK, Apekey TA, Cheung BM (2015). Gamma-glutamyltransferase and risk of hypertension: a systematic review and dose-response meta-analysis of prospective evidence. J Hypertens.

[40] Kunutsor SK, Apekey TA, Khan H (2014). Liver enzymes and risk of cardiovascular disease in the general population: a meta-analysis of prospective cohort studies. Atherosclerosis.

[41] Kunutsor SK, Blom AW, Whitehouse MR, Kehoe PG, Laukkanen JA (2017). Renin-angiotensin system inhibitors and risk of fractures: a prospective cohort study and meta-analysis of published observational cohort studies. Eur J Epidemiol.

[42] Kunutsor SK, Isiozor NM, Khan H, Laukkanen JA. Handgrip strength - a risk indicator for type 2 diabetes: systematic review and meta-analysis of observational cohort studies. Diabetes Metab Res Rev. 2020a:e3365. 10.1002/dmrr.3365. 10.1002/dmrr.3365 32543028

[43] Kunutsor SK, Khan H, Nyyssonen K, Laukkanen JA (2016). Lipoprotein(a) and risk of sudden cardiac death in middle-aged Finnish men: a new prospective cohort study. Int J Cardiol.

[44] Kunutsor SK, Khan H, Zaccardi F, Laukkanen T, Willeit P, Laukkanen JA (2018). Sauna bathing reduces the risk of stroke in Finnish men and women: a prospective cohort study. Neurology.

[45] Kunutsor SK, Laukkanen JA (2016). Serum zinc concentrations and incident hypertension: new findings from a population-based cohort study. J Hypertens.

[46] Kunutsor SK, Makikallio TH, Seidu S, de Araujo CGS, Dey RS, Blom AW, Laukkanen JA (2020). Physical activity and risk of venous thromboembolism: systematic review and meta-analysis of prospective cohort studies. Eur J Epidemiol.

[47] Kunutsor SK, Makikallio TH, Voutilainen A, Laukkanen JA. Handgrip strength is not associated with risk of venous thromboembolism: a prospective cohort study. Scand Cardiovasc J. 2020c:1–5. 10.1080/14017431.2020.1751267. 10.1080/14017431.2020.1751267 32281425

[48] Kunutsor SK, Voutilainen A, Laukkanen JA. Handgrip strength improves prediction of type 2 diabetes: a prospective cohort study. Ann Med. 2020 (In Press). 10.1080/07853890.2020.1815078 PMC7877957 32840381

[49] Kunutsor SK, Whitehouse MR, Blom AW, Laukkanen JA (2017). Low serum magnesium levels are associated with increased risk of fractures: a long-term prospective cohort study. Eur J Epidemiol.

[50] Laaksi IT, Ruohola JP, Ylikomi TJ, Auvinen A, Haataja RI, Pihlajamaki HK, Tuohimaa PJ (2006). Vitamin D fortification as public health policy: significant improvement in vitamin D status in young Finnish men. Eur J Clin Nutr.

[51] Laukkanen JA, Laaksonen D, Lakka TA, Savonen K, Rauramaa R, Makikallio T, Kurl S (2009). Determinants of cardiorespiratory fitness in men aged 42 to 60 years with and without cardiovascular disease. Am J Cardiol.

[52] Laukkanen JA, Voutilainen A, Kurl S, Araujo CGS, Jae SY, Kunutsor SK. Handgrip strength is inversely associated with fatal cardiovascular and all-cause mortality events. Ann Med. 2020a:1–11. 10.1080/07853890.2020.1748220. 10.1080/07853890.2020.1748220 PMC7877981 32223654

[53] Laukkanen JA, Voutilainen A, Kurl S, Isiozor NM, Jae SY, Kunutsor SK (2020). Handgrip strength is inversely associated with sudden cardiac death. Mayo Clin Proc.

[54] Leong DP (2015). Prognostic value of grip strength: findings from the Prospective Urban Rural Epidemiology (PURE) study. Lancet.

[55] Lynch JW, Kaplan GA, Cohen RD, Kauhanen J, Wilson TW, Smith NL, Salonen JT (1994). Childhood and adult socioeconomic status as predictors of mortality in Finland. Lancet.

[56] MacMahon S, Peto R, Cutler J, Collins R, Sorlie P, Neaton J, Abbott R, Godwin J, Dyer A, Stamler J (1990). Blood pressure, stroke, and coronary heart disease. Part 1, Prolonged differences in blood pressure: prospective observational studies corrected for the regression dilution bias. Lancet.

[57] Michaelsson K (2007). Leisure physical activity and the risk of fracture in men. PLoS Med.

[58] Moher D, Liberati A, Tetzlaff J, Altman DG (2009). Preferred reporting items for systematic reviews and meta-analyses: the PRISMA statement. PLoS Med.

[59] Phipps MM, et al. Acute liver injury in COVID-19: prevalence and association with clinical outcomes in a large US cohort. Hepatology. 2020. 10.1002/hep.31404. 10.1002/hep.31404 PMC7300739 32473607

[60] Piirtola M, Vahlberg T, Isoaho R, Aarnio P, Kivela SL (2008). Predictors of fractures among the aged: a population-based study with 12-year follow-up in a Finnish municipality. Aging Clin Exp Res.

[61] Pouresmaeili F, Kamalidehghan B, Kamarehei M, Goh YM (2018). A comprehensive overview on osteoporosis and its risk factors. Ther Clin Risk Manag.

[62] Prieto-Alhambra D, Turkiewicz A, Reyes C, Timpka S, Rosengren B, Englund M (2020). Smoking and alcohol intake but not muscle strength in young men increase fracture risk at middle age: a cohort study linked to the Swedish National Patient Registry. J Bone Miner Res.

[63] Rikkonen T, Poole K, Sirola J, Sund R, Honkanen R, Kroger H (2018). Long-term effects of functional impairment on fracture risk and mortality in postmenopausal women. Osteoporos Int.

[64] Robbins JA, Schott AM, Garnero P, Delmas PD, Hans D, Meunier PJ (2005). Risk factors for hip fracture in women with high BMD: EPIDOS study. Osteoporos Int.

[65] Rouzi AA, Al-Sibiani SA, Al-Senani NS, Radaddi RM, Ardawi MS (2012). Independent predictors of all osteoporosis-related fractures among healthy Saudi postmenopausal women: the CEOR Study. Bone.

[66] Salonen JT (1988). Is there a continuing need for longitudinal epidemiologic research? The Kuopio Ischaemic Heart Disease Risk Factor Study. Ann Clin Res.

[67] Savino E (2013). Handgrip strength predicts persistent walking recovery after hip fracture surgery. Am J Med.

[68] Selakovic I, Dubljanin-Raspopovic E, Markovic-Denic L, Marusic V, Cirkovic A, Kadija M, Tomanovic-Vujadinovic S, Tulic G (2019). Can early assessment of hand grip strength in older hip fracture patients predict functional outcome?. PLoS One.

[69] Sipers WM, Verdijk LB, Sipers SJ, Schols JM, van Loon LJ (2016). The Martin vigorimeter represents a reliable and more practical tool than the Jamar dynamometer to assess handgrip strength in the geriatric patient. J Am Med Dir Assoc.

[70] Sogaard AJ (2020). Grip strength in men and women aged 50-79 years is associated with non-vertebral osteoporotic fracture during 15 years follow-up: the Tromso Study 1994-1995. Osteoporos Int.

[71] Stattin K, Michaelsson K, Larsson SC, Wolk A, Byberg L (2017). Leisure-time physical activity and risk of fracture: a cohort study of 66,940 men and women. J Bone Miner Res.

[72] Stroup DF (2000). Meta-analysis of observational studies in epidemiology. JAMA.

[73] Tarantino U, Piccirilli E, Fantini M, Baldi J, Gasbarra E, Bei R (2015). Sarcopenia and fragility fractures: molecular and clinical evidence of the bone-muscle interaction. J Bone Joint Surg Am.

[74] Wickham CA, Walsh K, Cooper C, Barker DJ, Margetts BM, Morris J, Bruce SA (1989). Dietary calcium, physical activity, and risk of hip fracture: a prospective study. BMJ.

